# Supplementary material for: Development of a novel real-time polymerase chain reaction assay for the sensitive detection of Schistosoma japonicum in human stool
Source: PLoS Negl Trop Dis. 2021 Oct 25;15(10):e0009877. doi: 10.1371/journal.pntd.0009877 (PMC8568117; doi:10.1371/journal.pntd.0009877)
Supplement: Attachment — Submitted filename: Response to Reviewers_09.12.2021 (2).docx [file pntd.0009877.s012.docx]

Dear Editorial Board,

We hope this message finds you well. We would like to thank the reviewers for their insightful comments and constructive criticism. Your feedback helped us improve the quality of our paper, and we are appreciative of the effort and assistance. Please find detailed comments to your suggestions and comments below.

Sincerely,

Sara Halili

On behalf of all the authors

**Methods**

**Reviewer #1:**

The paper focuses on the analytical design and testing of a qPCR for S. japonicum. For Plos NTD the methods need more detail. Point details are shown below but a careful read and editing by the authors would help bring clarity to the methods and to provide the detail needed by the readers.

We thank the reviewer for this comment. We understand that providing additional detail regarding our methods will strengthen the clarity of our manuscript. We have supplied more information and details in the manuscript to address the suggestions below.

Page 7 – first paragraph. For the samples from the NHM – were these from worms or miracidia? If from worms why was WGA done? Also the geographical origin would be useful.

We thank the reviewer for this comment. The samples from the National History Museum (NHM) were obtained from single male worms collected from Zanzibar Island, Tanzania; Senegal (River Basin); and Hubei, China for *S. haematobium*, *S. mansoni*, and *S. japonicum*, respectively. This information has been added to the manuscript at lines 132-139 one pages 6-7. Whole genome amplification was performed because we did not have enough genomic DNA to carry out all the experiments needed for the assay’s optimization and validation.

Validation and Optimization – last sentence – which genomic DNA was used. ? S. japonicum.

We thank the reviewer for pointing out this need for clarification. *S. janonicum* genomic DNA was used to determine the limit of detection. This detail has been added in the manuscript.

Specificity section page 7 and 8 – the origin / supplier of the samples / DNA tested needs to be added.

We thank the reviewer for this comment. This information has been provided in (S1 Table), mentioned on page 8, line 164.

Plasmid control – add some more detail on how the target sequence was generated. PCR and then clean up etc.? How many repeats were in the target cloned. Also, a little more detail to this. How the colonies were selected, screened and purified etc.

We thank the reviewer for this comment, pointing out the need for additional detail. The SjTR1 qPCR forward and reverse primers were used to amplify the target sequence from genomic *S. japonicum* DNA using conventional PCR. The target sequence was size selected using agarose gel electrophoresis and was purified using the Monarch Genomic DNA Purification kit, and cloned using the Zero Blunt TOPO PCR Cloning Kit (Invitrogen, Carlsbad, CA) as previously described [26]. The plasmid was used to transform NEB Express Competent *E. coli* cells (New England Biolabs), which were plated on LB-kanamycin plates and grown overnight at 37°C. Following random selection, colony PCR and sequencing was performed. The plasmid containing a single copy of the assay’s target sequence was used as positive control and to calculate the assay’s efficiency. These details have been added to the manuscript on page 8, lines 166-175.

Explain more on how you go from a plasmid to the working concentration. This will not be common knowledge for many readers.

We thank the reviewer for this comment. The concentration of the plasmid was quantified using a Qubit 2.0 Fluorometer (Thermofisher Scientific, Waltham, MA) and diluted down to the desired working concentrations. The number of plasmid copies for each dilution was calculated with 100 ag of *S. japonicum* DNA corresponding to 26 copies of the target sequence. The data used to calculate the assay’s efficiency has been supplied in S6 Table on page 12 line 255 of the manuscript.

Testing naïve stool samples – where were the eggs from, state that they are S. japonicum and say how you counted / selected them. How was the weight of the stool measured and also I could not understand the addition of buffer to reserve room in the tube and was the whole 1g sample extracted?

We thank the reviewer for this comment. The eggs were obtained from the NIAID Schistosomiasis Resource Center of the Biomedical Research Institute (Rockville, MD) through NIH-NIAID contract HHSN2722010000051, which has been stated in the acknowledgements section on pages 20-21, lines 411-412. The manuscript has been altered to reflect that the eggs are *S. japonicum*, which was also reflected in the title of the section. The weight of the stool was measured using an accurate balance and the full 1g of stool was extracted. We modified the kit protocol slightly, adding less buffer prior to homogenization, because the volume of the tube was limiting. This has been clarified in the text on page 9, lines 194-197.

Figure 1 is confusing. You have highlighted the forward primer and the probe on the reverse strand. Although it is not incorrect it is confusing as I am not sure why you would not just highlight them on the forward strand and the reverse primer on the reverse strand? Also the two strands are not aligned so it does not make it easy to look at. Just have one strand in the forward direction and then mark the primers and probe and the sequence.

We thank the reviewer for this helpful comment. We understand how our figure was confusing and have changed the figure to reflect the suggestions, showing a single strand as well as the location of forward and reverse primers and probe.

**Reviewer #2:**

I feel that the number of species of gut microbes used in cross-reaction testing is insufficient. At a bare minimum, this must be tested against Schistosoma mekongi DNA. I understand that the authors have had difficulty obtaining this, but I encourage them to redouble their efforts. Researchers have published the transcriptome, are there at least in silico sequences that could be tested? I see this as a major deficiency in the paper.

We thank the reviewer for this comment. We acknowledge that not testing our assay on *S. mekongi* was a significant limitation. We were finally able to obtain *S. mekongi* worms from Dr. Poom Adisakwattana at Mahidol University in Thailand. After sequencing the 18S rRNA gene in the DNA isolated from the *S. mekongi* worms to confirm species identity, we tested *S. mekongi* gDNA and found that it was amplified by our assay. We also tested *T. solium*, *T. crassiceps*, *G.intestinalis*, and *N. americanus* none of which were amplified by our assay.

Further cross-reaction testing against Giardia duodenalis, a series of other Enterobacteriaceae, Enterococcus spp., Taenia spp., Necator americanus, Clonorchis sinensis and Opisthorchis viverrini, should be performed. DNA of the former five species/groups are easily obtained, so I am surprised that this has not already been performed.

We thank the reviewer for this comment. We were able to obtain DNA from and test our assay on *Necator americanus*, *Giardia intestinalis, Taenia solium, Taenia crassiceps*. In addition, the mock community B we tested contains a variety of species including *Acinetobacter baumannii, Actinomyces odontolyticus, Bacillus cereus, Bacteroides vulgatus, Clostridium beijerinckii, Deinococcus radiodurans, Enterococcus faecalis, Escherichia coli, Helicobacter pylori, Lactobacillus gasseri, Listeria monocytogenes, Neisseria meningitidis, Propionibacterium acnes, Pseudomonas aeruginosa, Rhodobacter sphaeroides, Staphylococcus aureus, Staphylococcus epidermidis, Streptococcus agalactiae, Streptococcus mutans, Streptococcus pneumoniae.*  Unfortunately, we were unable to obtain *Clonorchis sinensis* and *Opisthorchis viverrin*i.

As this assay may well be used on animals by other researchers, if possible, it should be tested on animal schistosomes as well, particularly S. malayensis of rats and S. incognitum of rats, dogs and pigs, both of which may cause rare human zoonotic infections in Asia.

We thank the reviewer for this comment. While we acknowledge it would be beneficial to test out assay on animal schistosomes, we were unfortunately unable to locate *S. malayensis* and *S. incognitum* despite our concerted and continuous efforts. If we are able to obtain these in the future, we will report this in a follow-up publication.

Where was the NHM S. japonicum DNA used from geographically? S. japonicum from Indonesia, China and the Philippines are quite genetically distinct. The Formosan (Taiwanese) strain of S. japonicum, which only infect animals, are genetically distant from other S. japnonicum. You must state the origin of your S. japonicum DNA and eggs used in this study.

We thank the reviewer for this comment. We have revised the manuscript to reflect the origin of *S. japonicum* DNA: Hubei, China on page 6 line 132. Again, we will work to obtain geographically distinct S. japonicum in the future and will report in a follow-up manuscript.

Reviewer #3: This manuscript describes a newly designed species-specific real-time PCR for the detection of Schistosoma japonicum DNA in human stool. The authors claim that by cleverly designing the right target (in this case: Schistosoma japonicum Tandem Repeat 1), their PCR is more sensitive than previously published real-time PCR assays for the detection of S. japonicum. I found the manuscript interesting to read and the PCR described seems to have great potential. My major criticism is that the authors did not use the opportunity to test their PCR on a collection of field samples. In my opinion this would have greatly benefitted the impact of the publication. I find it hard to imagine these authors have no access at all to a set of clinical stool samples, even if only a small one, to prove the diagnostic value of their PCR. A set of samples collected before and after treatment with praziquantel would have been ideal to illustrate the clinical value of their PCR.

We thank the reviewer for their insightful comments on our manuscript. We were indeed struggling to find field collected samples from endemic areas. This process was only made more difficult during the pandemic. However, after persistent efforts, we were able to obtain 100 clinical samples from collaborators at the QIMR Berghofer Medical Research Institute in Brisbane, Australia. This process was complicated due to a need for a CDC permit to import the samples to the United States, which significantly delayed our revision process. Given our assay's superior performance in testing clinical samples, we believe that it would be very successful for use in field studies and welcome any opportunities for collaboration to evaluate prevalence of infection in endemic areas prior to and following praziquantel mass drug administration treatment. We see this as an ideal follow-up publication.

Some minor points concerning methods:

Page 7: ” DNA was extracted from samples of S. japonicum, S. mansoni, and S. haematobium graciously provided by the Natural History Museum, London, UK” – more details about the original samples would have been informative.

We thank the reviewer for this helpful comment. We have updated the manuscript to reflect the origin of the *Schistosoma* worms from the Natural History Museum of London on page 6, lines 132-133.

Page 8 bottom: I would like to read more details of the source of the S. japonicum eggs. Do these eggs fully represent the eggs normally seen in freshly collected stool samples?

We thank the reviewer for this comment. *S. japonicum* eggs were provided by the NIAID Schistosomiasis Resource Center of the Biomedical Research Institute (Rockville, MD) through NIH-NIAID contract HHSN2722010000051. To assess the effectiveness of the SjTR1 real-time PCR assay on field-collected samples, we compared the SjTR1 assay to Kato-Katz and the NADH dehydrogenase PCR assay on 100 clinical samples collected from an *S. japonicum* endemic region in the Philippines.

**Results**

Reviewer #1:

The results need some detail added and also need a careful read to make sure nothing is missing. Also as required by Plos NTD all qC values should be provided for all replicates and all samples, even the controls.

We thank the reviewer for this comment. We have provided the data in multiple supplemental files containing each replicate for all of the data discussed in the manuscript.

Describe what happened at temperatures above and below 59.

We thank the reviewer for this comment. We initially tested a range of temperatures from 52 ℃ to 62 ℃ at 2 ℃ degree increments (52 ℃, 54 ℃, 56 ℃, 58 ℃, 60 ℃, 62 ℃) where 58 ℃ yielded the lowest Cq value. To get more specific results, we tested a few 0.5 ℃ degree increments (56.5 ℃, 57 ℃, 57.5 ℃, 58 ℃, 58.5 ℃, 59℃) where 59℃ resulted in the lowest Cq value and was used as the optimized annealing temperature. This data has been provided in a supplemental table (S2 Table) and the additional detail is provided in the manuscript on page 7, lines 144-148.

Was there a cut off Cq score for non-amplification used to test the LOD and the specificity? You do not mention S. mansoni or S. haematobium in the specificity section.

We thank the reviewer for this comment. The species reported as non-amplified in the specificity section had undetermined Cq values after 40 cycles. Normally, we consider a Cq of 40 or less as positive. The manuscript has been modified to reflect that *S. mansoni* and *S. haematobium* were *not* amplified by our assay on page 11 line 245.

It would be better to refer to the published targets that you tested as published targets rather than reference targets and make it clear that these are published and not designed as part of this study.

We thank the reviewer for this helpful suggestion. The manuscript has been modified to reflect this change.

You mention the negative controls in the results but all the negative controls and how they were set up need to be described in the methods. Negative controls also need to be described for the validation steps.

We thank the reviewer for this comment. More detail has been provided regarding the negative control in the validation and spiking experiments. No eggs were spiked in naive human stool for the negative control. Negative controls in our study contain the same master mix/reagents used in the rest of the experiment without template DNA or eggs added.

**Conclusions**

Reviewer #1:

Although the discussion supports the data and the study it is very brief and does not address the wider context of the study. Suggestions for improvement are shown below.

Line three change “historically occurred” to “typically performed” as you should refer to what is typically done now.

We thank the reviewer for this helpful comment. The change has been made in the manuscript on page 2 line 29, page 3 line 55, page 4 line 83, and page 17 line 324.

It would be good to provide some information on the interventions in S. japonicum areas. Is routine mass testing done or is it done on a case by case basis, is MDA performed or is it a test and treat scenario. The wider context for the use of qPCR as a test needs to be discussed particularly in relation to other tests such as serology and antigen. What would be the application of a qPCR test in these settings?

We thank the reviewer for this comment. In S*. japonicum*-endemic areas, preventive chemotherapy with praziquantel is used. Eligibility of different age groups for treatment as well as intensity of implementation of treatment is determined based on infection prevalence. Thus, accurate diagnostic methods are necessary for the successful elimination of *S. japonicum* from endemic areas. This information has been provided in the discussion section on page 17, lines 325-343.

Page 15 – when talking about the critical need for specificity you could include the recent Plod NTD paper by Katie Gass.

We thank the reviewer for bringing this impactful paper to our attention. The paper has been included in the discussion section on page 17 line 343.

When talking about the need to look at field samples you should also mention other variables such as would the presence of CFPD have an impact?

We thank the reviewer for the comment. We acknowledge that in certain cases, like determining success of mass drug treatment efforts, a test’s ability to distinguish between CFPD and egg derived DNA may be important, as CFPD might be present for a while following treatment. That is beyond the scope of our study.

You should also talk about the endemic setting and how such a qPCR could fit. For example in S. japonicum settings are labs available for the testing or are portable systems needed like in Africa and also some discussion around cost is needed. Does the test need to fit TPP’s of schisto?

We thank the reviewer for this insightful comment. *S. japonicum* is endemic in the Philippines, China, Indonesia, and Taiwan. Due to HIV and more recently COVID testing, there are many real-time PCR testing labs in the endemic areas. Furthermore, even in settings where PCR testing is not currently available, it is important that these tools are established. In addition, there are a variety of advances made yearly that are bringing the cost of PCR down. Based on a previous study by our lab, the cost of a PCR duplicate testing of a sample and a double-slide Kato-Katz are comparable (about two US dollars). This has been added in the discussion section of the manuscript on page 20 lines 396-401.

You should also talk about the diagnostic in terms of intensity analysis – limitations or if it can be used as a quantitative assay or not?

We thank the reviewer for this comment. While our assay isn’t yet designed as a quantitative assay, qPCR is semiquantitative by nature. It will take additional work in the future to evaluate the use of PCR as a means of measuring intensity of infection. Because we are not using the assay quantitatively in this paper, we have changed the title to “real-time” to reflect this limitation.

Also, talk about the potential utility in animal and snail hosts.

We thank the reviewer for this comment. Although testing in animal and snail hosts would be beneficial given the wide range of animal hosts that *S. japonicum* infects, we have not conducted testing yet on snails and animals. Again, we plan to do such work for future publications.

**Reviewer #2:**

The greatest issue with the discussion as it stands is the attempt to minimise the need for further validation of this assay in an endemic setting on both human and animal samples. This must be amended and the deficiencies of not having done this very clearly and openly acknowledged and explored.

We thank the reviewer for this comment. We did not intend to downplay the importance of further validation of our assay in clinical samples from human and animal subjects. We had originally intended to do this in a follow-up publication. Based on these and other comments, we obtained a set of clinical samples and now report these data in the manuscript. We also have amended our manuscript to better reflect the limitations of our work.

Discussion first sentence: Also endemic in Taiwan (zoophilic Formosan strain)

We thank the reviewer for bringing this to our attention. The manuscript has been amended to reflect this change on page 3 line 67 and page 17 line 322.

If the authors do not test against the many genotypes of S. japonicum found in Asia, they must clearly state that their assay described here has not been validated against the multiple genotypes possible and that this work should be performed in the future.

We thank the reviewer for this comment. We have acknowledged this limitation in our discussion on page 18 line 362-363 and we plan to perform this type of optimization in a future study.

Similarly, if the authors do not test against the S. japonicum from multiple animal hosts and multiple human hosts, they have not entirely validated their assay and they must state such testing is required to validate the assay before any clinical, public health and particularly veterinary use.

We thank the reviewer for this comment. While use of the test for screening of animal hosts would be important for accurate assessment of infection prevalence given that *S. japonicum* has a wide range of animal hosts, in this paper we assess the suitability of the assay for detection of *S. japonicum* in human stool. To support this purpose, we validated our assay using 100 human clinical stool samples from the Philippines. Again, we acknowledge the limitation of not testing our assay on clinical samples from all the areas where *S. japonicum* is endemic but the only samples we were able to obtain in a reasonable timeframe were collected in the Philippines. We intend, in the future, to obtain samples through collaboration from other regions and from animal hosts.

Page 16 first para. I do not believe that the spiking of S. japonicum eggs into naive human stool samples provides a close approximation of real samples since our mock samples. The point of such validations is to test multiple isolates from different hosts as well as to approximate its validity in the faecal matrix. Please significantly amend this sentence to reflect my comment here.

We thank the reviewer for this comment. We have amended the sentence per your suggestion. What we meant is that the stool samples we spiked with eggs contained possible PCR inhibitors that could impact our assay’s efficiency. We did not mean to suggest that such testing is sufficient for final validation of the assay on samples from different regions and from different animal hosts.

Page 16 para 1: Overall, this assay needs to be compared to Kato Katz and some of the other PCRs against a statistically valid number of human and animal samples in an endemic setting, preferably three endemic settings (one in Indonesia, one in China and one in Indonesia). I understand that this work may yet be performed, but I think it is important that the authors better acknowledge the need for such validation very clearly in the discussion, please amend the sentence addressing the need for field validation to better.

We thank the reviewer for this comment. We have amended the discussion section to clearly address the limitations of our work as well as possibilities for future exploration. We tested our assay and the NADH dehydrogenase I assay against 100 human stool samples collected from endemic areas in the Philippines. Again, we acknowledge the limitation of not having tested our assay on animal host stool samples or human clinical samples from all the areas where *S. japonicum* is endemic but the only samples we were able to obtain within a reasonable timeframe were samples collected in the Philippines. We plan to test our assay on clinical samples from more human and animal hosts from different regions when available.

Line 15: I see the failure to test the assay against S. mekongi as a very significant limitation. You should remove the sentence saying that it is not and acknowledge that validation is not complete without such testing.

We thank the reviewer for this comment. We acknowledge that not having tested our assay on *S. mekongi* was a significant limitation and have now performed the suggested testing on genomic DNA isolated from *S. mekongi* worms supplied to us from Mahidol University in Salaya, Thailand. The results from such testing are displayed on page 12 lines 246-247 and discussed on page 18 lines 351-363 in the manuscript.

Reviewer #3: As mentioned above, I find the overall content of this manuscript rather limited. As the authors have mentioned, the focus is entirely on the technical characteristics of the PCR, without any clinical validation. This is a serious omission. One of the major advantages of S. japonicum is the fact that this parasite also affects various non-human hosts, such a cattle. So even if the authors have no access to human stool, they could have tested naturally infected animal samples.

We thank the reviewer for this comment. We made serious efforts to find clinical samples in endemic areas and have been able to obtain a significant number of human stool samples collected in the Philippines to perform further validation of our assay. Unfortunately, we have not yet been able to obtain clinical samples from animal hosts and this limitation has been acknowledged in the discussion on page 20 line 391-393. We intend to further explore the effectiveness of the assay against a variety of samples from different countries and different animals in our follow-up work. Getting this assay published will make it much easier to obtain such samples from collaborators.

Another limitation of the manuscript is that the authors suggest throughout the manuscript that PCR is the only diagnostic alternative to microscopic detection of S. japonicum eggs. In fact, detection of circulating antigen in serum or urine, a more field-friendly procedure for diagnosing an active S. japonicum infection, has also shown to be a promising alternative. See: Van Dam et al., (2015) Evaluation of banked urine samples for the detection of circulating anodic and cathodic antigens in Schistosoma mekongi and S. japonicum infections: a proof-of-concept study. Acta Trop. 2015 Jan;141(Pt B):198-203.

We thank the reviewer for this comment. We apologize for making it sound as if PCR is the only diagnostic alternative for detection of *S. japonicum*. We have altered the manuscript’s discussion to make it clear that there are other options. The point of presenting a highly sensitive qPCR test is to begin work to provide a gold standard against which other assays can be compared in the future.

**Editorial and Data Presentation Modifications**

Reviewer #1:

A careful read and careful revision of terminology would help improve the paper. Some sections are hard to follow and could be written better and with more detail to help interpretation.

We thank the reviewer for this comment. We have made all the suggested corrections to improve the clarity of our writing.

Reviewer #2:

The results section is very "table heavy" – this could be remedied by changing table 3 into a figure.

We thank the reviewer for this comment. We have modified table 3 into a figure. Due to a concentration error, testing of the mitochondrial 16S rRNA gene target was repeated and the updated data were used to create figure 3 on page 13 lines 281-282 while the initial data are shown in table 3 on page 14 of the marked up manuscript.

page 12 first para - no space between genus and species name for "S. japonicum"

We thank the reviewer for this comment. The change has been made to the manuscript.

Page 15 final para - italicise S. mekongi

We thank the reviewer for this comment. The manuscript has been amended to reflect this.

**Summary and General Comments**

Reviewer #1:

This is a very informative paper that describes the development and lab. testing of a S. japonicum qPCR assay to support diagnosis. This will be a very useful assay due to it superior sensitivity. The way the target was identified and used shows substantial progress in the design of molecular assays and the use of genomic data that is now becoming available. This will also support the design of assays for other species. The paper needs more detail and a more wider discussion to bring it up to the standard required for Plos NTD.

We thank the reviewer for these generous comments. We have added more detail and expanded our discussion to better reflect the limitations of our work.

Some points on the author summary and intro are shown below.

Please add line numbers as this helps with reviewing.

We thank the reviewer for this comment and apologize for the lack of line numbers in our initial draft.

Author Summary – 230 million is for schisto total not for S. japonicum – revise the first sentence. This should also be made clear in the first line of the introduction.

We thank the reviewer for bringing this mistake to our attention. We have made the appropriate changes in the manuscript on page 3 lines 52-54 and lines 65-68 as we understand how the sentence structure was indeed confusing.

Page 4 last paragraph – add stool or faecal before samples.

We thank the reviewer for this comment. The change has been made on page 4 line 83.

Line 5 state if this is egg derived DNA or CFPD or both.

We thank the reviewer for their comment. Real-time PCR is able to detect DNA regardless of the source so we used the term “parasite-derived DNA” to include both egg derived DNA and CFPD.

Reviewer #2: This is a worthwhile assay but further validation against other common gut parasites found in S. japonicum endemic regions is needed.

We thank the reviewer for this generous comment. We have further tested our assay against *S. mekongi*, *T. solium*, *T. cracisepps*, *G. intestinalis*, and *N.americanus*, of which only the most closely related species, *S. mekongi,* was amplified. Our assay is very sensitive in detection of *S. mekong*i gDNA, but the suitability of our assay for detection of *S. mekongi* will be explored in a future study. Although these results clearly indicate a limitation for our assay’s specificity, the fact that *S. japonicum* and *S. mekong*i are only co-endemic in a limited region in Myanmar gives us confidence that the assay could be used for the differential detection of both species in endemic areas where they’re not co-endemic. In the regions where co-endemicity is an issue, molecular differentiation could be achieved via sequencing of the variable 18S RNA gene. This has been addressed in the discussion section on page 18 lines 351-363.

Furthermore, if S. mekongi DNA cannot be obtained, or sequences analysed in silico, this must be acknowledged as a significant deficiency.

We thank the reviewer for this suggestion. We were able to test our assay on DNA isolated from *S. mekongi* worms, supplied by the Applied Malacology Laboratory, Faculty of Tropical Medicine, Mahidol University, Thailand. Our assay did amplify *S. mekongi* gDNA and this limitation is addressed in the discussion section.

The authors tend to use terms such as "we do not feel this is a significant limitation" when describing deficiencies of their own work. This not only gets a reviewer's "back up" as it is not for the authors to decide what is an is not a significant limitation of their approach, it is also not good scientific writing. I suggest simply acknowledging your work's deficiencies without attempting to editorialize on how important or unimportant they are.

We thank the reviewer for this comment. We have adjusted our discussion to better reflect the limitations of our work.

Reviewer #3:

This manuscript is well written, the objectives are clear and the PCR described is potentially relevant for those working in the field of S. japonicum. Nevertheless, the study would benefit from some additional experimental work, in particular testing of real clinical samples.

We thank the reviewer for this generous comment! We acknowledge that validation in clinical samples is very important and thus performed additional testing on field collected samples from the Philippines, provided to us from QIMR Berghofer Medical Research Institute in Australia.
