# Supplementary material for: Development of a novel real-time polymerase chain reaction assay for the sensitive detection of Schistosoma japonicum in human stool
Source: PLoS Negl Trop Dis. 2021 Oct 25;15(10):e0009877. doi: 10.1371/journal.pntd.0009877 (PMC8568117; doi:10.1371/journal.pntd.0009877)
Supplement: Attachment — Submitted filename: PNTD-D-21-00356R2_Response to Reviewers .docx [file pntd.0009877.s013.docx]

Dear Editorial Board,

We hope this message finds you well. We would like to thank the reviewers for their helpful suggestions. Please find detailed comments to your suggestions and comments below.

Sincerely,

Sara Halili

On behalf of all the authors

Reviewer #2:

The authors have now tested their assay against S. mekongi, which was my major concern upon review of the original manuscript. They have addressed my other comments well.

We thank the reviewer for the comment. Their thoughtful suggestions helped us improve the quality of our initial manuscript.

While the reviewers have now included the zoophilic S. japonicum from Taiwan, it has not been acknowledged as only infecting animals. I would like to suggest the authors include a sentence noting this. I think it is important, as otherwise an error may enter the literature with this article being cited by the uninformed as describing human infection from/in Taiwan.

We thank the reviewer for bringing this issue to our attention. The manuscript has been edited to reflect that the strain in Taiwan is zoophilic in lines 67 and 322.
